# Endoscopic ultrasound-guided drainage of a right liver fistula caused by percutaneous thermoablation

**DOI:** 10.1055/a-2641-1807

**Published:** 2025-07-25

**Authors:** Angelica Toppeta, Jean-Philippe Ratone, Solene Hoibian, Yanis Dahel, Antoine Assaf, Marc Giovannini, Fabrice Caillol

**Affiliations:** 1 Endoscopy Unit, Paoli Calmettes Institute, Marseille, France; 2 472674 Endoscopy Unit, ASST Fatebenefratelli Sacco, Milan, Italy

We present a case of a 35-year-old man who developed a biloma in the right liver following thermoablation for liver metastasis. His medical history included distal splenopancreatectomy for grade 2 neuroendocrine tumors, followed by left hepatectomy for liver metastasis 5 years before. The patient recently underwent chemotherapy and a few sessions of percutaneous thermoablation for liver metastases. The biloma was treated via percutaneous drainage ( Fig. 1 ). However, after noting that the biloma was only partially resolved, a biliary fistula was identified. Endoscopic retrograde cholangiopancreatography was attempted, but the bile ducts were atrophied; thus, cannulation of the target bile duct failed. Neither sphincterotomy nor plastic stent placement in the right liver resolved the fistula. After multidisciplinary discussions, we opted to internalize the biliary fistula via endoscopic ultrasound (EUS)-guided drainage. This approach was selected based on research indicating that the right liver is accessible via EUS 1 2 3 . A curved linear therapeutic EUS-scope was introduced into the duodenum under fluoroscopy. Contrast and saline solution were injected into the fistula through a percutaneous drain to delineate the trajectory under EUS and fluoroscopy ( Fig. 2 ). The fistula was punctured from the duodenum using a 19-G needle, and a 0.025-in guidewire was inserted. The tract was first dilated using a 6-Fr cystotome and then a 4-mm biliary balloon catheter. A double pigtail stent was inserted into the fistula and duodenum. Minor bleeding occurred but ceased spontaneously. Contrast was injected via the percutaneous drain, and successful drainage of the contrast agent into the duodenum confirmed correct placement of the stent ( Fig. 3 ). One week later, a second double pigtail stent was placed parallel to the first ( Video 1 ). The percutaneous drain was removed 48 hours later. This video shows a valid EUS-guided alternative for internalizing a biliary fistula from the duodenum when standard methods fail.

**Fig. 1:**
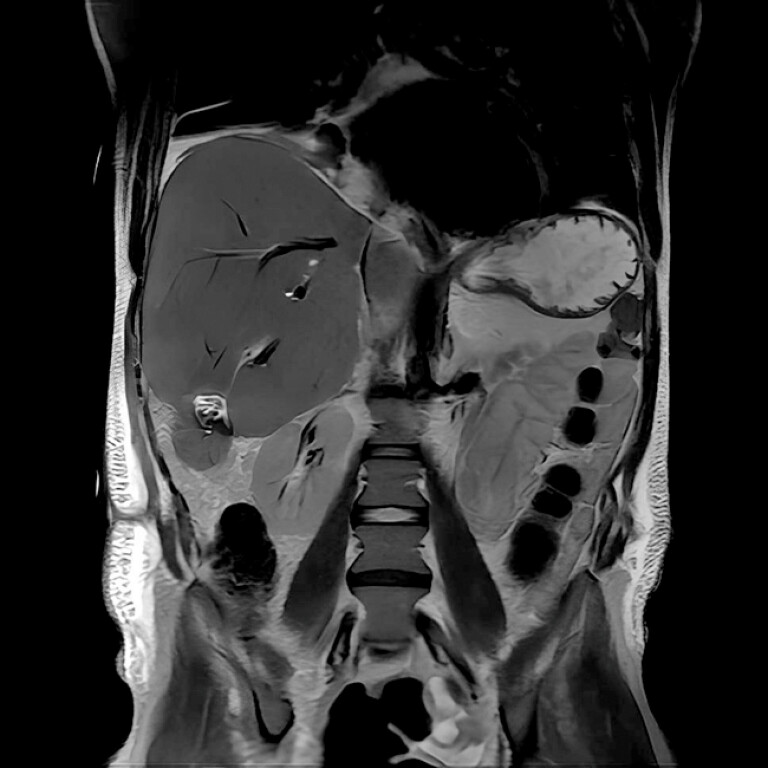
IRM image showing a biliary fistula in the right hepatic lobe and biloma.

**Fig. 2:**
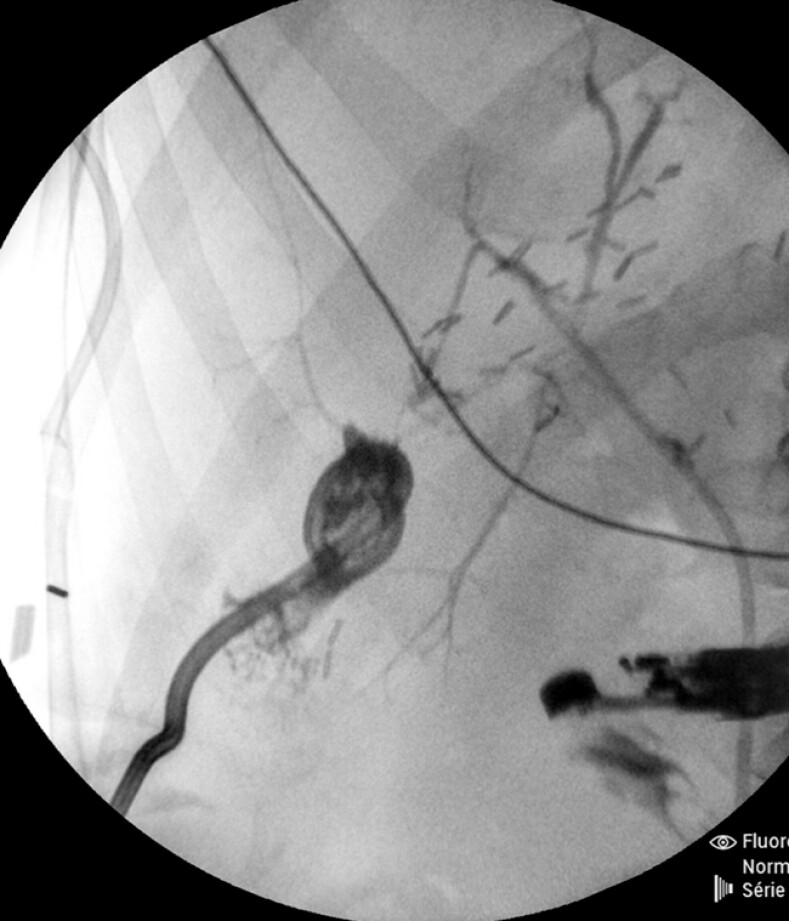
Fluoroscopy image of the biliary fistula after contrast agent injection via the percutaneous drain.

**Fig. 3:**
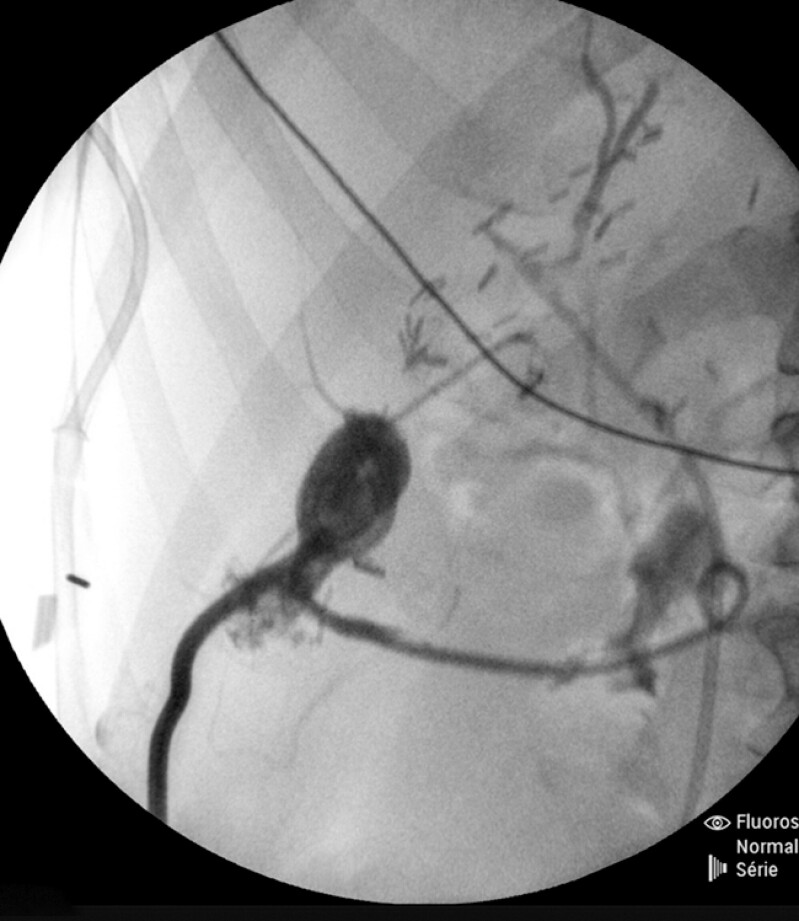
Fluoroscopy image showing contrast medium draining into the duodenum.

EUS-guided drainage of a right liver fistula from the duodenum with two double pigtail stents.Video 1

Endoscopy_UCTN_Code_TTT_1AS_2AH

## Notes

Endoscopy E-Videos https://eref.thieme.de/e-videos
*E-Videos* is an open access online section of the journal *Endoscopy* , reporting on interesting cases and new techniques in gastroenterological endoscopy. All papers include a high-quality video and are published with a Creative Commons CC-BY license. Endoscopy E-Videos qualify for HINARI discounts and waivers and eligibility is automatically checked during the submission process. We grant 100% waivers to articles whose corresponding authors are based in Group A countries and 50% waivers to those who are based in Group B countries as classified by Research4Life (see: https://www.research4life.org/access/eligibility/ ). This section has its own submission website at https://mc.manuscriptcentral.com/e-videos .
